# Developing a quantitative model of antibody effector function

**DOI:** 10.1186/1742-4690-9-S2-P175

**Published:** 2012-09-13

**Authors:** AW Boesch, M Ackerman

**Affiliations:** 1 Thayer School of Engineering at Dartmouth, Hanover, NH, USA

## Background

While the induction of neutralizing antibodies remains a cornerstone of HIV vaccine development, the success of the Thai trail and evidence from non-human primate (NHP) studies has indicated that non-neutralizing antibodies may provide protection from infection. These non-neutralizing antibodies can act as molecular beacons to recruit innate immune cells to recognize infected CD4T cells or free virus as pathogenic. The ability of an antibody to efficiently recruit innate immune cells depends on complex interactions between the pathogenic target, the antibody, and an effector cell.

## Methods

Here we present a quantitative model of natural killer cell mediated antibody dependent cellular cytotoxicity (ADCC), and examine the impact of core fucosylation, FcgR3a expression/occupancy, serum IgG competition and mutations in the Fc binding domain.

## Results

The results indicate that FcgR3a site occupancy can predict the percentage of cells killed on an ADCC dose response curve. In addition, it appears ADCC is initiated past a threshold of FgR3a site occupancy on the natural killer cell.

## Conclusion

This provides key insight into the mechanism of the innate immune response and provides powerful evidence that the effector function of antibodies should be a key consideration in the development of HIV vaccines.

